# Краткий обзор клинических руководств Европейского кардиологического общества 2023 года по лечению сердечно-сосудистых заболеваний у пациентов с сахарным диабетом

**DOI:** 10.14341/probl13414

**Published:** 2024-09-16

**Authors:** A. Д. Эрлих, A. В. Зилов, Д. Ю. Щекочихин, С. Б. Шорников, Е. В. Бублик, О. И. Виноградская, А. И. Гришковец, А. Г. Фарманов, Е. Г. Рыжкова

**Affiliations:** Ильинская больница; Ильинская больница; Первый Московский государственный медицинский университет им. И.М. Сеченова (Сеченовский Университет); Ильинская больница; Первый Московский государственный медицинский университет им. И.М. Сеченова (Сеченовский Университет); Ильинская больница; Ильинская больница; Ильинская больница; Ильинская больница; Ильинская больница; Первый Московский государственный медицинский университет им. И.М. Сеченова (Сеченовский Университет); Ильинская больница; Первый Московский государственный медицинский университет им. И.М. Сеченова (Сеченовский Университет)

**Keywords:** сахарный диабет, сердечно-сосудистые заболевания, Европейское кардиологическое общество

## Abstract

Широкая распространенность и опасность сердечно-сосудистых заболеваний (ССЗ) хорошо известна. По данным Всемирной Организации Здравоохранения (ВОЗ), от ССЗ в мире ежегодно умирает почти 18 миллионов человек, что составляет 31% от всех причин смерти [1].

Во многих случаях ССЗ протекают в тесной ассоциации с сахарным диабетом (СД), а повышенный уровень глюкозы крови является причиной около 20% смертей от ССЗ [2]. При этом среди причин смерти у пациентов с СД 2 типа (СД2) превалируют именно ССЗ. Так, по данным Федерального регистра СД 2022 г., в России непосредственной причиной смерти у пациентов с СД2 в 24,2% случаев была хроническая сердечная недостаточность, в 13,1% случаев — острая сердечная недостаточность, в 10,0% случаев — нарушение мозгового кровообращения, в 3,7% случаев — инфаркт миокарда [3]. Тесная патофизиологическая связь атеросклеротических ССЗ и СД закономерно привела к тому, что в клинической практике кардиологов все больше встречается пациентов с диабетом, а в практике эндокринологов — пациентов с ССЗ. Эта связь стала столь очевидной, что в недавней заметке, размещенной в Европейском кардиологическом журнале, Ю. Браунвальд рассуждал о появлении новой медицинской специальности — диабетокардиологии [4]. К сожалению, прогнозы экспертов показывают, что число пациентов с диабетом на планете достигнет 783 миллионов [5].

Важной причиной для необходимости объединения подходов в лечении ССЗ и СД стало появление в последнее время доказательств об эффективности определенных классов сахароснижающих препаратов в отношении сердечно-сосудистых исходов. Это касается в первую очередь некоторых ингибиторов рецепторов SGLT2, а также ряда агонистов ГПП-1, и нового неселективного антагониста минералокортикоидных рецепторов финеренона.

Именно поэтому, учитывая важность объединенных и скоординированных усилий по ведению пациентов с ССЗ и СД, рабочая группа экспертов Европейского кардиологического общества (ЕКО) в 2023 г. обновила, сформировала и опубликовала документ — Клинические руководства по лечению ССЗ у пациентов с СД [6]. Настоящий материал будет посвящен краткому обзору основных положений этого документа.

## Abstract (en)

The prevalence of cardiovascular diseases (CVDs) is well known. According to the World Health Organization (WHO), almost 18 million people die from CVDs worldwide every year, accounting for 31% of all causes of death [1]. CVDs often develop concomitantly with diabetes mellitus (DM), with approximately 20% of cardiovascular deaths attributed to elevated blood glucose levels [2]. Notably, CVDs are the leading cause of death among patients with type 2 diabetes (T2DM). Based on data from the Federal Register of Diabetes 2022 in Russia, chronic heart failure was the direct cause of death in 24.2% of T2DM cases, followed by acute heart failure (13.1%), cerebrovascular events (10.0%), and myocardial infarction (3.7%) [3].

The pathophysiological interplay between atherosclerotic cardiovascular disease and DM has led to a situation where cardiologists are increasingly involved in the treatment of patients with DM, while endocrinologists are encountering a growing number of patients with CVDs. This association has become so apparent that in a recent article published in the European Journal of Cardiology, Yu. Braunwald speculated about the emergence of a new subspecialty - diabetocardiology [4]. Unfortunately, experts predict that the global number of diabetic patients will reach 783 million [5].

Recent data on the CV benefits of certain hypoglycemic drugs (primarily, certain SGLT2 inhibitors, several GLP-1 receptor agonists, and a novel non-steroidal mineralocorticoid receptor antagonist finerenone) prove the need for a unified interdisciplinary approach to managing CVDs and DM.

Given the importance of integrated and coordinated efforts in managing patients with CVD and DM, the Task Force of the ­European Society of Cardiology (ESC) updated, formulated, and published clinical guidelines on the treatment of CVD in diabetic patients in 2023 [6]. This article provides a concise overview of the key provisions outlined in the guidelines.

Клинические руководства рекомендуют проводить обязательный скрининг на наличие СД (рекомендация класса IA) всем пациентам с ССЗ, включая лиц с фибрилляцией предсердий (ФП) и сердечной недостаточностью (СН). В отношении установления диагноза «СД» традиционно используются тесты с определением глюкозы в крови натощак (не менее 8 часов без приема пищи), через 2 часа после глюкозной нагрузки (пероральный прием 75 г глюкозы), случайное определение глюкозы и оценка уровня гликированного гемоглобина (HbA1c). Для определения уровня глюкозы натощак ее повышение ≥7,0 ммоль/л (≥126 мг/дл) является диагностическим признаком диабета (обычно одного теста достаточно для пациентов с симптомами гипергликемии, а при отсутствии симптомов диагноз подтверждается после второго теста).

Также наличие СД подтверждается уровнем гликемии через 2 часа после глюкозной нагрузки 11,1 ммоль/л и выше или уровнем HbA1c 6,5% и выше (рис. 1).

**Figure:**
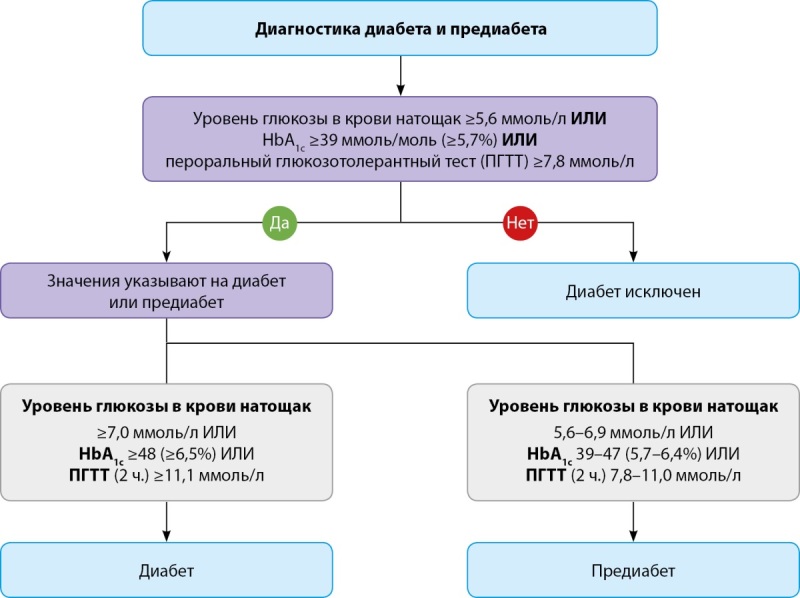
Рисунок 1. Диагностика диабета и предиабета (заимствовано и адаптировано из [6]).

Наоборот, всем пациентам с СД2, у которых еще нет атеросклеротического ССЗ (АССЗ) или нет выраженного поражения органов-мишеней (ПОМ), рекомендована оценка риска развития ССЗ с использованием шкалы SCORE2-Диабет (класс IB). В нее, помимо традиционных факторов риска, которые используются в шкале SCORE2, входят показатели, связанные с СД: длительность заболевания, уровень HbA1c, скорость клубочковой фильтрации (СКФ) [7]. Оценка степени выраженности ПОМ (выявление ХБП, альбуминурии, ретинопатии, наличие АССЗ) также является обязательной для пациента с СД (класс IB).

## СЕРДЕЧНО-СОСУДИСТЫЕ РИСКИ У ПАЦИЕНТОВ С СД: ЦЕЛИ И ЛЕЧЕНИЕ

Авторы документа подчеркивают огромную значимость изменения образа жизни и утверждают, что у пациентов с СД2 именно модификация образа жизни является ключевой и обязательной для снижения риска ССЗ. В качестве рекомендации класса IA говорится, что лицам с ожирением или избытком массы тела нужно снижать вес и повышать физические нагрузки. Другие лечебные воздействия также могут быть использованы, но имеют меньшую значимость: использование агонистов рецепторов ГПП-1 или бариатрическая хирургия (класс IIaB).

В отношении оптимизации питания пациентам с СД2 для снижения риска ССЗ рекомендовано (класс IA) использовать адаптированную средиземноморскую или растительную диету (пищевой рацион, богатый усвояемыми пищевыми волокнами) с высоким содержанием ненасыщенных жиров.

Эксперты также рекомендуют обязательное увеличение физической активности (хотя бы в виде 10-минутной пешей прогулки), упоминая, что оптимальная умеренно-интенсивная нагрузка должна длиться 150 мин, а интенсивная — 75 минут в неделю (IA), и говоря о необходимости выполнения нагрузок с сопротивлением по крайней мере 2 раза в неделю (IB). Для пациентов с уже имеющимся ССЗ, в том числе с ишемической болезнью сердца (ИБС), любым типом СН, ФП, рекомендовано использовать структурированные физические упражнения, то есть разделение занятий на отдельные сегменты и предварительное их планирование (IB).

Всем курящим пациентам с СД2 для снижения риска ССЗ показано обязательное прекращение курения (IA). В качестве возможной помощи для достижения этой цели необходимо рассмотреть использование никотин-заместительной терапии, применение варениклина или бупропиона, а также индивидуальные или дистанционные консультации (IIaB).

Рекомендован контроль артериального давления (желательны измерение АД на каждом визите и использование гипотензивных препаратов) при офисном АД≥140/90 мм рт.ст. (IA). Целевые значения АД при лечении гипертонии у пациентов с СД рекомендовано определять индивидуально и стремиться к систолическому АД <130 мм рт.ст., если это хорошо переносится, но при этом избегать снижения <120 мм рт.ст., а для лиц старше 65 лет целевым САД может считаться значение 130–139 мм рт.ст. (IA).

Важнейшая часть рекомендаций — коррекция нарушений липидного спектра у пациентов с СД. Эксперты подтвердили прежние «европейские» целевые значения в отношении холестерина ЛПНП: для пациентов с умеренным риском ССЗ — <2,6 ммоль/л (IA), высоким риском — <1,8 ммоль/л и снижение ЛПНП по крайней мере на 50% от исходного (IA), а при очень высоком риске — <1,4 ммоль/л и снижение ЛПНП по крайней мере на 50% от исходного (IB). Медикаментозными препаратами первой линии для достижения целевых значений ЛПНП у пациентов с СД являются статины (IA), а в случае, если максимальные или максимально переносимые дозы статина не позволяют достичь целевых значений, рекомендовано добавление эзетемиба (IB). Пациентам с очень высоким сердечно-сосудистым риском со стабильно высоким уровнем ЛПНП, выше целевого несмотря на прием максимально переносимой дозы статина, в сочетании с эзетимибом или пациентам с непереносимостью статинов, рекомендовано назначение ингибитора PCSK9 (IA).

## КОНТРОЛЬ ГЛИКЕМИИ: ЦЕЛИ И СПОСОБЫ ИХ ДОСТИЖЕНИЯ

Вопрос целевого уровня гликемии у пациентов с СД довольно сложный, так как, с одной стороны, данные исследований показывают связь более интенсивного лечения с лучшими микроваскулярными исходами (в отношении нейро- и ретинопатии) [8], с другой стороны, более низкие целевые уровни HbA1c могут быть ассоциированы с повышением смертности от сердечно-сосудистых причин [9]. Поэтому, формулируя рекомендации по целевым уровням гликемии, экспертная группа ЕКО была очень осторожна в заключениях.

Можно выделить следующие ключевые положения:

Эксперты указывают, что для снижения отдаленного риска ИБС следует предпочесть более строгий контроль гликемии, а в лечении желательно отдавать предпочтение препаратам с доказанной сердечно-сосудистой пользой [IIaB].

Опираясь на накопившиеся данные рандомизированных исследований, эксперты ЕКО утверждают, что выбор гипогликемической терапии должен быть в первую очередь основан на преимуществах в предотвращении ССЗ, которые это лечение принесет. Поэтому назначение ингибитора SGLT2 или агониста ГПП-1 для пациентов с СД должно быть также обязательно, как и, например, назначение статина или ингибитора АПФ/блокатора АТ-рецепторов. Для пациентов с СД и имеющимся АССЗ назначение этих препаратов отнесено к классу I (рис. 2).

**Figure:**
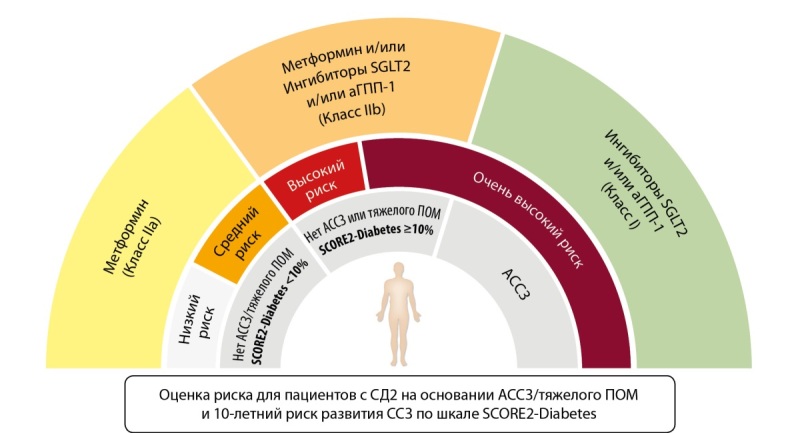
Рисунок 2. Выбор сахароснижающей терапии у пациентов с СД2 на основе наличия АССЗ, степени СС-риска, поражения органов-мишеней (заимствовано и адаптировано из [6]).

Важным аспектом является также то, что у пациентов с СД2 и известным АССЗ лечение должно начаться с агониста ГПП-1 и/или ингибитора SGLT2 — препаратов с доказанной эффективностью снижения риска ССЗ — независимо от уровня HbA1c и сопутствующего приема сахароснижающих препаратов (класс I) (рис. 3).

**Figure:**
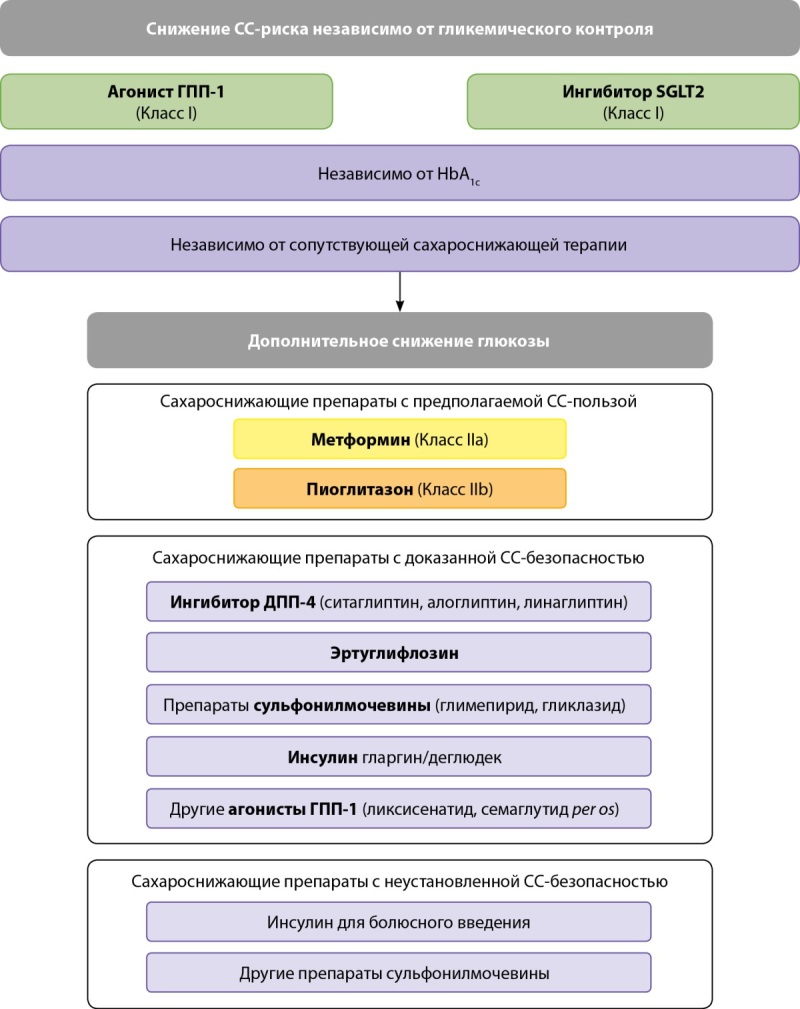
Рисунок 3. Схема сахароснижающей терапии у пациентов с СД2 и АССЗ для снижения сердечно-сосудистого риска(заимствовано и адаптировано из [6]).

Для улучшения гликемического контроля эксперты предлагают рассмотреть возможность добавления метформина (IIa) и пиоглитазона (IIb).

## АНТИТРОМБОТИЧЕСКАЯ ТЕРАПИЯ

В отношении использования антиагрегантов, антикоагулянтов или их сочетания у пациентов с СД2 положения обновленных клинических руководств мало изменились по сравнению с предыдущими регламентирующими документами. Так, например, эксперты указывают на возможность обсудить использование ацетилсалициловой кислоты (АСК) в низкой дозе (75–100 мг) у пациентов с СД2 для первичной профилактики, если к этому нет явных противопоказаний (IIbA). Такими противопоказаниями считается желудочно-кишечное кровотечение или язва желудка в предыдущие 6 мес, активная болезнь печени (цирроз или активный гепатит) или непереносимость АСК.

Программа ASCEND [10], где изучалось использование АСК в крупном РКИ в отношении предотвращения ССЗ и которая, строго говоря, была единственным крупным и прямым рандомизированным исследованием, посвященным этой теме, показала преимущество АСК над плацебо. Однако оно проявлялось в первые 3 года лечения, а в последующие годы различий в исходах между группами АСК и плацебо не было, частота больших кровотечений (в основном желудочно-кишечных) на фоне АСК была значимо выше, а NNT и NNH для АСК против плацебо почти не различались, и были 91 и 111 соответственно.

Небольшие изменения получила рекомендация об использовании ингибиторов протонной помпы (ИПП) в сочетании с антитромботическими препаратами. Указано, что любая комбинация антитромботических средств требует обязательного одновременного использования ИПП для профилактики желудочно-кишечных кровотечений (класс IA). При этом эксперты не рекомендуют использовать клопидогрел одновременно с омепразолом и эзомепразолом (класс IIIB), и это положение вызывает вопросы. Во-первых, потому что работа, которая стала основанием для этой рекомендации, была лишь анализом медицинских баз данных, а во-вторых, потому что она показала, что повышенный риск сердечно-сосудистых осложнений встречался при сочетании клопидогрела с омепразолом, ланзопразолом, эзомепразолом и пантопразолом, но не рабепразолом [11].

## ОСТРЫЙ КОРОНАРНЫЙ СИНДРОМ У ПАЦИЕНТОВ С СД

Проблема коррекции гликемии у пациентов с острым коронарным синдромом (ОКС) всегда была актуальной, а результаты нескольких исследований с противоположными результатами оставляют этот вопрос скорее открытым, чем решенным. Большинство имеющихся данных говорят о том, что наилучшей тактикой в отношении гликемии в первые часы ОКС является умеренно строгий контроль (стремление к целевым показателями 10–11 ммоль/л), при возможности избегая гипогликемии. Такой подход, например, был связан с лучшими исходами по сравнению с более жестким контролем гликемии в исследовании у пациентов с тяжелыми заболеваниями в реанимационных отделениях NICE-SHUGAR [12]. При этом авторы Клинических руководств подчеркивают, что у всех пациентов с ОКС обязательно нужно оценить исходный гликемический статус (класс IB) и часто его мониторировать, выявляя гипергликемию, то есть уровень глюкозы выше 11,0 ммоль/л (класс IC), а также рассмотреть необходимость использования сахароснижающей терапии, избегая гипогликемии (класс IIaC). В отношении того, как именно нужно корректировать уровень глюкозы крови при ОКС, детально не указывается, но в большинстве случаев при значительной гипергликемии предлагается использовать инфузию инсулина. Хотя недавнее исследование EMMY показало, что применение эмпаглифлозина в ранние сроки острого инфаркта миокарда может быть связано с улучшением уровня NT-proBNP и функции левого желудочка [13].

## СЕРДЕЧНАЯ НЕДОСТАТОЧНОСТЬ И ДИАБЕТ

За последние годы наиболее масштабные изменения в лечении ССЗ коснулись именно лечения пациентов с сердечной недостаточностью (СН), и именно «на стыке» с лечением СД. Речь идет о препаратах из группы ингибиторов SGLT2 (дапаглифлозине и эмпаглифлозине), которые уже несколько лет являются препаратами «первой линии» в лечении пациентов с СН и сниженной фракцией выброса левого желудочка (ФВЛЖ) [14]. Более поздние исследования показали возможность улучшения исходов, связанную с приемом эмпаглифлозина [15] и дапаглифлозина [16] у пациентов с сохранной ФВЛЖ. Тот факт, что изначально сахароснижающие препараты из группы ингибиторов SGLT2 у пациентов с СН эффективны вне зависимости от наличия или отсутствия СД, лишает положения рекомендаций по лечению СН специфичности для пациентов с СД.

В отношении же лечения СД у пациентов с СН или риском развития СН эксперты рекомендуют назначать в первую очередь препараты с доказанной пользой в отношении СС-исходов (класс IA) либо при необходимости менять сахароснижающую терапию, переходя от нейтральных или потенциально опасных в отношении СС-исходов препаратов к тем, для которых доказана СС-польза (класс IC).

Ключевые положения руководств указывают на обязательное использование одного из ингибиторов SGLT2 (дапаглифлозина или эмпаглифлозина) или ингибитора SGLT2/1 сотаглифлозина (препарат пока не зарегистрирован в РФ) у пациентов с СН и низкой ФВЛЖ (класс IA), а также на обязательное использование эмпаглифлозина или дапаглифлозина у пациентов с СН и ФВЛЖ>40% (класс IA).

В отношении других групп сахароснижающих препаратов эксперты ЕКО считают возможным использование агонистов ГПП-1 (ликсисенатид, лираглутид, семаглутид и др.), как и ингибиторов ДПП-4 (ситаглиптин и линаглиптин), не влияющих на риски СН и ее осложнений у пациентов с СД, имеющих СН или риск развития СН (класс IIaA). Схожие рекомендации даны в отношении использования метформина и базальных инсулинов (гларгин и деглудек) (класс IIaB).

К сахароснижающим препаратам, которые не рекомендованы у пациентов с СН или риском развития СН, эксперты отнесли производное тиазолидиндиона пиоглитазон, а также ингибитор ДПП-4 саксаглиптин из-за повышенного риска развития СН и госпитализации из-за СН у пациентов с СД2 (класс IIIB). В отношении саксаглиптина основанием для нежелательности его использования у пациентов с риском СН стали результаты исследования SAVOR-TIMI-53, где анализ вторичных конечных точек показал связь препарата с большим риском развития СН, что особенно бросалось в глаза на фоне отсутствия какой-либо прогностической пользы [17].

На рис. 4 схематично представлены общие принципы применения сахароснижающей терапии у пациентов с СН и СД2.

**Figure:**
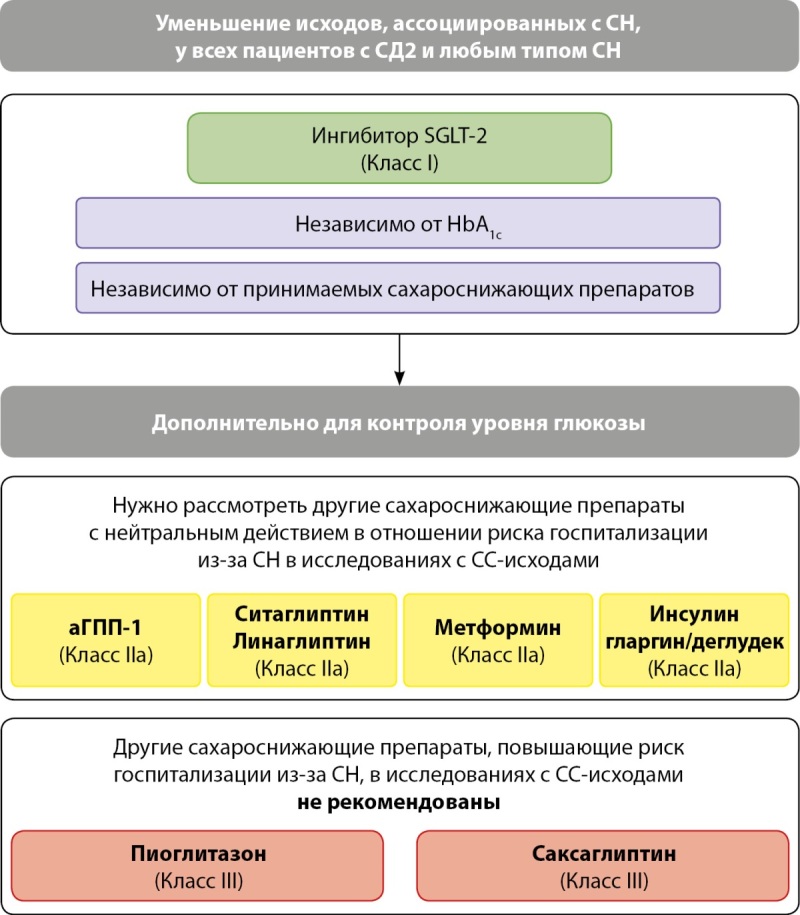
Рисунок 4. Сахароснижающая терапия у пациентов с СН и СД2 (заимствовано и адаптировано из [6]).

## ФИБРИЛЛЯЦИЯ ПРЕДСЕРДИЙ И ДИАБЕТ

Строго говоря, эксперты ЕКО не дают каких-либо специфических рекомендаций в отношении ведения пациентов с СД и фибрилляцией предсердий (ФП). Однако, с учетом того, что наличие СД является фактором риска тромбоэмболических осложнений и компонентом шкалы CHADS-VAsc, предлагается рассмотреть использование орального антикоагулянта для профилактики инсульта у пациентов с ФП и СД, но без других факторов тромбоэмболического риска (класс IIaB).

## ХРОНИЧЕСКАЯ БОЛЕЗНЬ ПОЧЕК И ДИАБЕТ

Функциональное нарушение почек является важной проблемой для пациентов с СД, так как почки, с одной стороны, являются одним из «органов-мишеней», а с другой — поражение почек (в том числе в связи с СД) связано с большим риском развитий ССЗ [18][19]. Поэтому в тексте Клинических руководств акцент в лечении диабета делается не на коррекцию гликемии как таковой, а на снижение рисков развития ССЗ и хронической болезни почек (ХБП).

Пациентам с СД рекомендовано проводить рутинный скрининг функции почек с оценкой СКФ по критериям CKD-EPI и оценивать альбумин-креатининовое соотношение в моче (класс IВ).

В рекомендациях для пациентов с СД и ХБП эксперты ЕКО указывают на обязательную интенсивную гиполипидемическую терапию статинами или сочетанием статин+эзетемиб, что особенно важно с учетом того, что ХБП является самостоятельным фактором риска развития ССЗ (класс IA). Хотя для пациентов с тяжелой ХБП на диализе польза от интенсивной гиполипидемической терапии менее очевидна.

Подчеркнута необходимость строгого контроля за уровнем АД с достижением его целевых значений не выше 130/80 мм рт.ст. для снижения риска ССЗ и альбуминурии (класс IA).

Эксперты указывают на индивидуальный выбор целевого уровня HbA1c — от 6,5 до 8,0%, но с желательным достижением уровня HbA1c<7,0% для снижения риска микрососудистых осложнений (класс IA).

Отмечается обязательное использование максимально переносимых доз ингибитора АПФ или блокатора АТ-рецепторов (класс IA), а всем пациентам с СД2 и ХБП с СКФ≥20 мл/мин/1,73 м² необходимо назначение ингибитора SGLT2 (канаглифлозин, эмпаглифлозин или дапаглифлозин) для снижения риска ССЗ и почечной недостаточности (класс IA). Для достижения адекватного контроля гликемии, снижения риска гипогликемии и пользы от снижения массы тела, снижения риска развития ССЗ и альбуминурии у пациентов с СКФ выше 15 мл/мин/1,73 м² эксперты рекомендуют использовать агонисты ГПП-1 (класс IA) (рис. 5).

**Figure:**
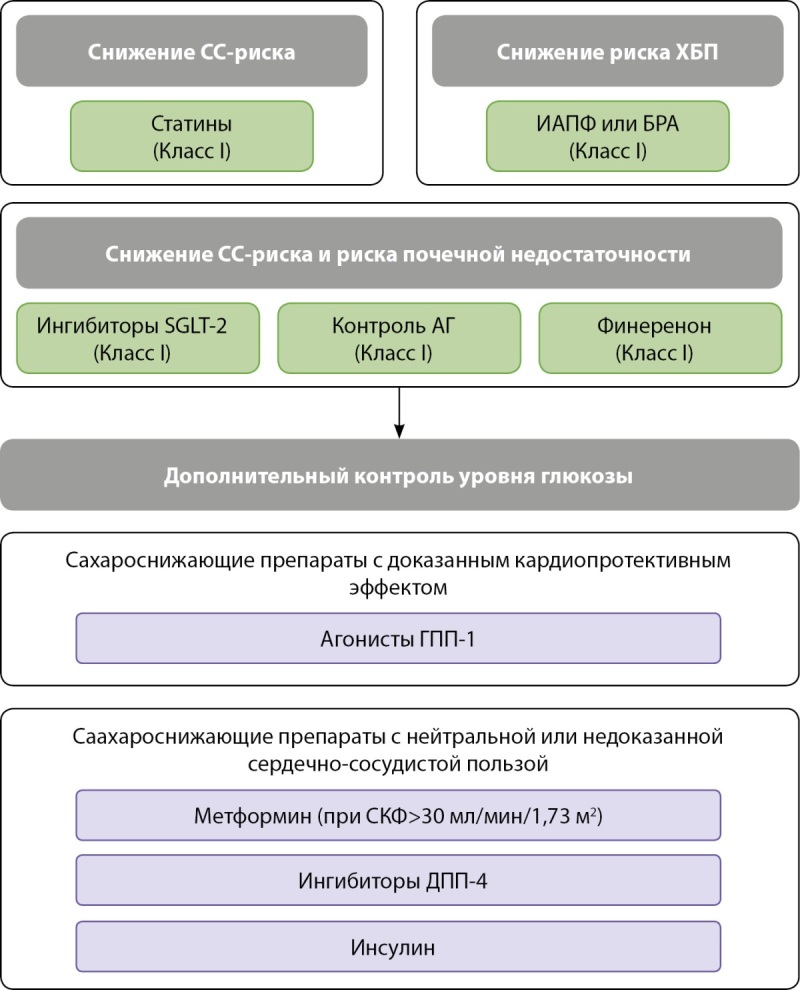
Рисунок 5. Медикаментозное ведение пациентов с СД2 и ХБП для снижения сердечно-сосудистого риска или риска почечной недостаточности (заимствовано и адаптировано из [6]).

Одно из новшеств этих руководств — препарат финеренон, использование которого рекомендовано экспертами (класс IA) в дополнение к ингибитору АПФ/блокатору АТ-рецепторов у пациентов с ХБП и альбуминурией (повышенное альбумин-креатининовое соотношение в моче) для снижения риска ССЗ и почечной недостаточности.

Финеренон является новым нестероидным селективным антагонистом минералокортикоидных рецепторов. В двойном слепом исследовании FIDELIO-DKD у >5,5 тыс пациентов с СД2 и ХБП, рандомизированных к приему финеренона или плацебо, за 2,5 года частота неблагоприятных событий (ухудшение функции почек в виде снижения СКФ≥40% и смерть от почечных причин) на фоне приема финеренона была значимо меньше: 17,8 vs. 21,1% (относительный риск 0,82; 95% доверительный интервал 0,73–0,93; р=0,001) [20].

Для пациентов с СД, ХБП и ИБС эксперты ЕКО рекомендуют в равной степени использовать консервативную тактику с оптимальной медикаментозной терапией или исходную инвазивную тактику из-за их неразличимых исходов (класс IB).

## ПАЦИЕНТООРИЕНТИРОВАННЫЙ ПОДХОД

Такая стратегия лечения предполагает не только полное информирование пациентов в отношении выбора лечения и принятия решений, но также помогает расширить возможности пациентов активно участвовать в поиске решений их проблем.

В рамках этого подхода рекомендовано предоставлять пациентам с СД структурированные образовательные программы для улучшения знаний о диабете, контроля гликемии, предотвращения осложнений, о правах и возможностях пациентов (класс IC). Также рекомендовано принимать решения в контексте целей и приоритетов пациента (класс IC).

## ЗАКЛЮЧЕНИЕ

В заключение обзора Клинических руководств ЕКО по лечению ССЗ у пациентов с СД надо сказать, что эта проблема остается актуальной и требует одновременного участия в судьбе одного пациента нескольких специалистов. Помимо кардиолога и эндокринолога, в эту работу нередко оказываются вовлечены эндоваскулярные хирурги, нефрологи, специалисты по питанию и реабилитации. В любом случае важно понимать, что в ежедневной клинической практике более строгое следование клиническим руководствам всегда ассоциировано с лучшими исходами. Тем более, что в новых Руководствах есть положения, основанные на доказательствах улучшения прогноза, что особенно важно для клиницистов и практикующих врачей.

## ДОПОЛНИТЕЛЬНАЯ ИНФОРМАЦИЯ

Источники финансирования. Работа выполнена по инициативе авторов без привлечения финансирования.

Конфликт интересов. Авторы декларируют отсутствие явных и потенциальных конфликтов интересов, связанных с содержанием настоящей статьи.

Участие авторов. Все авторы одобрили финальную версию статьи перед публикацией, выразили согласие нести ответственность за все аспекты работы, подразумевающую надлежащее изучение и решение вопросов, связанных с точностью или добросовестностью любой части работы.

## References

[cit1] Vsemirnaya Organizatsiya Zdravookhraneniya (Internet). Serdechno-sosudistye zabolevaniya. Dostupno po: https://www.who.int/ru/health-topics/cardiovascular-diseases#tab=tab_1

[cit2] Global Burden of Disease Collaborative Network. Global Burden of Disease Study 2019. Results. Institute for Health Metrics and Evaluation. 2020. Available from: https://www.healthdata.org/research-analysis/gbd

[cit3] Dedov I. I., Shestakova M. V., Vikulova O. K., Zheleznyakova A. V., Isakov M. A., Sazonova D. V., Mokrysheva N. G. (2023). Diabetes mellitus in the Russian Federation: dynamics of epidemiological indicators according to the Federal Register of Diabetes Mellitus for the period 2010–2022. Diabetes mellitus.

[cit4] Braunwald Eugene (2023). Diabetocardiology: a new subspecialty?. European Heart Journal.

[cit5] Sun Hong, Saeedi Pouya, Karuranga Suvi, Pinkepank Moritz, Ogurtsova Katherine, Duncan Bruce B., Stein Caroline, Basit Abdul, Chan Juliana C.N., Mbanya Jean Claude, Pavkov Meda E., Ramachandaran Ambady, Wild Sarah H., James Steven, Herman William H., Zhang Ping, Bommer Christian, Kuo Shihchen, Boyko Edward J., Magliano Dianna J. (2021). IDF Diabetes Atlas: Global, regional and country-level diabetes prevalence estimates for 2021 and projections for 2045. Diabetes Research and Clinical Practice.

[cit6] Marx Nikolaus, Federici Massimo, Schütt Katharina, Müller-Wieland Dirk, Ajjan Ramzi A, Antunes Manuel J, Christodorescu Ruxandra M, Crawford Carolyn, Di Angelantonio Emanuele, Eliasson Björn, Espinola-Klein Christine, Fauchier Laurent, Halle Martin, Herrington William G, Kautzky-Willer Alexandra, Lambrinou Ekaterini, Lesiak Maciej, Lettino Maddalena, McGuire Darren K, Mullens Wilfried, Rocca Bianca, Sattar Naveed, Prescott Eva, Cosentino Francesco, Abdelhamid Magdy, Aboyans Victor, Antoniou Sotiris, Asteggiano Riccardo, Baumgartner Iris, Buccheri Sergio, Bueno Hector, Čelutkienė Jelena, Chieffo Alaide, Christersson Christina, Coats Andrew, Cosyns Bernard, Czerny Martin, Deaton Christi, Falk Volkmar, Ference Brian A, Filippatos Gerasimos, Fisher Miles, Huikuri Heikki, Ibanez Borja, Jaarsma Tiny, James Stefan, Khunti Kamlesh, Køber Lars, Koskinas Konstantinos C, Lewis Basil S, Løchen Maja-Lisa, McEvoy John William, Mihaylova Borislava, Mindham Richard, Neubeck Lis, Nielsen Jens Cosedis, Parati Gianfranco, Pasquet Agnes A, Patrono Carlo, Petersen Steffen E, Piepoli Massimo Francesco, Rakisheva Amina, Rossello Xavier, Rossing Peter, Rydén Lars, Standl Eberhard, Tokgozoglu Lale, Touyz Rhian M, Visseren Frank, Volpe Massimo, Vrints Christiaan, Witkowski Adam, Hazarapetyan Lusine, Zirlik Andreas, Rustamova Yasmin, van de Borne Philippe, Sokolović Šekib, Gotcheva Nina, Milicic Davor, Agathangelou Petros, Vrablík Michal, Schou Morten, Hasan-Ali Hosam, Viigimaa Margus, Lautamäki Riikka, Aboyans Victor, Klimiashvili Zurab, Kelm Malte, Siasos Gerasimos, Kiss Róbert Gábor, Libungan Berglind, Durkan Maeve, Zafrir Barak, Colivicchi Furio, Tundybayeva Meiramgul, Bytyçi Ibadete, Mirrakhimov Erkin, Trusinskis Karlis, Saadé Georges, Badarienė Jolita, Banu Cristiana-Astra, Magri Caroline Jane, Boskovic Aneta, Hattaoui Mustapha El, Martens Fabrice, Bosevski Marijan, Knudsen Eva Cecilie, Burchardt Paweł, Fontes-Carvalho Ricardo, Vinereanu Dragos, Mancini Tatiana, Beleslin Branko, Martinka Emil, Fras Zlatko, Conde Almudena Castro, Mellbin Linda, Carballo David, Bsata Walid, Mghaieth Fathia, Gungor Baris, Mitchenko Olena, Wheatcroft Stephen, Trigulova Raisa, Prescott Eva, James Stefan, Arbelo Elena, Baigent Colin, Borger Michael A, Buccheri Sergio, Ibanez Borja, Køber Lars, Koskinas Konstantinos C, McEvoy John William, Mihaylova Borislava, Mindham Richard, Neubeck Lis, Nielsen Jens Cosedis, Pasquet Agnes A, Rakisheva Amina, Rocca Bianca, Rosselló Xavier, Vaartjes Ilonca, Vrints Christiaan, Witkowski Adam, Zeppenfeld Katja (2023). 2023 ESC Guidelines for the management of cardiovascular disease in patients with diabetes. European Heart Journal.

[cit7] Pennells Lisa, Kaptoge Stephen, Østergaard Helena Bleken, Read Stephanie H, Carinci Fabrizio, Franch-Nadal Josep, Petitjean Carmen, Taylor Owen, Hageman Steven H J, Xu Zhe, Shi Fanchao, Spackman Sarah, Gualdi Stefano, Holman Naomi, Da Providencia E Costa Rui Bebiano, Bonnet Fabrice, Brenner Hermann, Gillum Richard F, Kiechl Stefan, Lawlor Deborah A, Potier Louis, Schöttker Ben, Sofat Reecha, Völzke Henry, Willeit Johann, Baltane Zane, Fava Stephen, Janos Sandor, Lavens Astrid, Pildava Santa, Poljicanin Tamara, Pristas Ivan, Rossing Peter, Sascha Reiff, Scheidt-Nave Christa, Stotl Iztok, Tibor Gail, Urbančič-Rovan Vilma, Vanherwegen An-Sofie, Vistisen Dorte, Du Yong, Walker Matthew R, Willeit Peter, Ference Brian, De Bacquer Dirk, Halle Martin, Huculeci Radu, McEvoy John William, Timmis Adam, Vardas Panagiotis, Dorresteijn Jannick A N, Graham Ian, Wood Angela, Eliasson Björn, Herrington William, Danesh John, Mauricio Dídac, Benedetti Massimo Massi, Sattar Naveed, Visseren Frank L J, Wild Sarah, Di Angelantonio Emanuele, Balkau Beverley, Bonnet Fabrice, Fumeron Frederic, Stocker Hannah, Holleczek Bernd, Schipf Sabine, Schmidt Carsten Oliver, Dörr Marcus, Tilg Herbert, Leitner Christoph, Notdurfter Marlene, Taylor Julie, Dale Caroline, Prieto-Merino David, Gillum Richard F, Lavens Astrid, Vanherwegen An-Sofie, Poljicanin Tamara, Pristas Ivan, Buble Tamara, Ivanko Pero, Rossing Peter, Carstensen Bendix, Heidemann Christin, Du Yong, Scheidt-Nave Christa, Gall Tibor, Sandor Janos, Baltane Zane, Pildava Santa, Lepiksone Jana, Magri Caroline J, Azzopardi Joseph, Stotl Iztok, Real Jordi, Vlacho Bogdan, Mata-Cases Manel (2023). SCORE2-Diabetes: 10-year cardiovascular risk estimation in type 2 diabetes in Europe. European Heart Journal.

[cit8] (2008). Intensive Blood Glucose Control and Vascular Outcomes in Patients with Type 2 Diabetes. New England Journal of Medicine.

[cit9] Ismail-Beigi Faramarz, Craven Timothy, Banerji Mary Ann, Basile Jan, Calles Jorge, Cohen Robert M, Cuddihy Robert, Cushman William C, Genuth Saul, Grimm Richard H, Hamilton Bruce P, Hoogwerf Byron, Karl Diane, Katz Lois, Krikorian Armand, O'Connor Patrick, Pop-Busui Rodica, Schubart Ulrich, Simmons Debra, Taylor Harris, Thomas Abraham, Weiss Daniel, Hramiak Irene (2010). Effect of intensive treatment of hyperglycaemia on microvascular outcomes in type 2 diabetes: an analysis of the ACCORD randomised trial. The Lancet.

[cit10] (2018). Effects of Aspirin for Primary Prevention in Persons with Diabetes Mellitus. New England Journal of Medicine.

[cit11] Niu Q, Wang Z, Zhang Y, et al. Combination use of clopidogrel and proton pump inhibitors increases major adverse cardiovascular events in patients with coronary artery disease: A meta-analysis. J Cardiovasc Pharmacol Therap. 2017;22(2):142–152. doi: https://doi.org/10.1177/1074248416663 10.1177/1074248416663647 27512080

[cit12] (2009). Intensive versus Conventional Glucose Control in Critically Ill Patients. New England Journal of Medicine.

[cit13] von Lewinski Dirk, Kolesnik Ewald, Tripolt Norbert J, Pferschy Peter N, Benedikt Martin, Wallner Markus, Alber Hannes, Berger Rudolf, Lichtenauer Michael, Saely Christoph H, Moertl Deddo, Auersperg Pia, Reiter Christian, Rieder Thomas, Siller-Matula Jolanta M, Gager Gloria M, Hasun Matthias, Weidinger Franz, Pieber Thomas R, Zechner Peter M, Herrmann Markus, Zirlik Andreas, Holman Rury R, Oulhaj Abderrahim, Sourij Harald (2022). Empagliflozin in acute myocardial infarction: the EMMY trial. European Heart Journal.

[cit14] McDonagh Theresa A, Metra Marco, Adamo Marianna, Gardner Roy S, Baumbach Andreas, Böhm Michael, Burri Haran, Butler Javed, Čelutkienė Jelena, Chioncel Ovidiu, Cleland John G F, Coats Andrew J S, Crespo-Leiro Maria G, Farmakis Dimitrios, Gilard Martine, Heymans Stephane, Hoes Arno W, Jaarsma Tiny, Jankowska Ewa A, Lainscak Mitja, Lam Carolyn S P, Lyon Alexander R, McMurray John J V, Mebazaa Alexandre, Mindham Richard, Muneretto Claudio, Francesco Piepoli Massimo, Price Susanna, Rosano Giuseppe M C, Ruschitzka Frank, Kathrine Skibelund Anne, de Boer Rudolf A, Christian Schulze P, Abdelhamid Magdy, Aboyans Victor, Adamopoulos Stamatis, Anker Stefan D, Arbelo Elena, Asteggiano Riccardo, Bauersachs Johann, Bayes-Genis Antoni, Borger Michael A, Budts Werner, Cikes Maja, Damman Kevin, Delgado Victoria, Dendale Paul, Dilaveris Polychronis, Drexel Heinz, Ezekowitz Justin, Falk Volkmar, Fauchier Laurent, Filippatos Gerasimos, Fraser Alan, Frey Norbert, Gale Chris P, Gustafsson Finn, Harris Julie, Iung Bernard, Janssens Stefan, Jessup Mariell, Konradi Aleksandra, Kotecha Dipak, Lambrinou Ekaterini, Lancellotti Patrizio, Landmesser Ulf, Leclercq Christophe, Lewis Basil S, Leyva Francisco, Linhart Aleš, Løchen Maja-Lisa, Lund Lars H, Mancini Donna, Masip Josep, Milicic Davor, Mueller Christian, Nef Holger, Nielsen Jens-Cosedis, Neubeck Lis, Noutsias Michel, Petersen Steffen E, Sonia Petronio Anna, Ponikowski Piotr, Prescott Eva, Rakisheva Amina, Richter Dimitrios J, Schlyakhto Evgeny, Seferovic Petar, Senni Michele, Sitges Marta, Sousa-Uva Miguel, Tocchetti Carlo G, Touyz Rhian M, Tschoepe Carsten, Waltenberger Johannes, Adamo Marianna, Baumbach Andreas, Böhm Michael, Burri Haran, Čelutkienė Jelena, Chioncel Ovidiu, Cleland John G F, Coats Andrew J S, Crespo-Leiro Maria G, Farmakis Dimitrios, Gardner Roy S, Gilard Martine, Heymans Stephane, Hoes Arno W, Jaarsma Tiny, Jankowska Ewa A, Lainscak Mitja, Lam Carolyn S P, Lyon Alexander R, McMurray John J V, Mebazaa Alexandre, Mindham Richard, Muneretto Claudio, Piepoli Massimo Francesco, Price Susanna, Rosano Giuseppe M C, Ruschitzka Frank, Skibelund Anne Kathrine (2021). 2021 ESC Guidelines for the diagnosis and treatment of acute and chronic heart failure. European Heart Journal.

[cit15] Anker Stefan D., Butler Javed, Filippatos Gerasimos, Ferreira João P., Bocchi Edimar, Böhm Michael, Brunner–La Rocca Hans-Peter, Choi Dong-Ju, Chopra Vijay, Chuquiure-Valenzuela Eduardo, Giannetti Nadia, Gomez-Mesa Juan Esteban, Janssens Stefan, Januzzi James L., Gonzalez-Juanatey Jose R., Merkely Bela, Nicholls Stephen J., Perrone Sergio V., Piña Ileana L., Ponikowski Piotr, Senni Michele, Sim David, Spinar Jindrich, Squire Iain, Taddei Stefano, Tsutsui Hiroyuki, Verma Subodh, Vinereanu Dragos, Zhang Jian, Carson Peter, Lam Carolyn Su Ping, Marx Nikolaus, Zeller Cordula, Sattar Naveed, Jamal Waheed, Schnaidt Sven, Schnee Janet M., Brueckmann Martina, Pocock Stuart J., Zannad Faiez, Packer Milton (2021). Empagliflozin in Heart Failure with a Preserved Ejection Fraction. New England Journal of Medicine.

[cit16] Solomon Scott D., McMurray John J.V., Claggett Brian, de Boer Rudolf A., DeMets David, Hernandez Adrian F., Inzucchi Silvio E., Kosiborod Mikhail N., Lam Carolyn S.P., Martinez Felipe, Shah Sanjiv J., Desai Akshay S., Jhund Pardeep S., Belohlavek Jan, Chiang Chern-En, Borleffs C. Jan Willem, Comin-Colet Josep, Dobreanu Dan, Drozdz Jaroslaw, Fang James C., Alcocer-Gamba Marco Antonio, Al Habeeb Waleed, Han Yaling, Cabrera Honorio Jose Walter, Janssens Stefan P., Katova Tzvetana, Kitakaze Masafumi, Merkely Béla, O’Meara Eileen, Saraiva Jose Francisco Kerr, Tereshchenko Sergey N., Thierer Jorge, Vaduganathan Muthiah, Vardeny Orly, Verma Subodh, Pham Vinh Nguyen, Wilderäng Ulrica, Zaozerska Natalia, Bachus Erasmus, Lindholm Daniel, Petersson Magnus, Langkilde Anna Maria (2022). Dapagliflozin in Heart Failure with Mildly Reduced or Preserved Ejection Fraction. New England Journal of Medicine.

[cit17] Scirica Benjamin M., Bhatt Deepak L., Braunwald Eugene, Steg P. Gabriel, Davidson Jaime, Hirshberg Boaz, Ohman Peter, Frederich Robert, Wiviott Stephen D., Hoffman Elaine B., Cavender Matthew A., Udell Jacob A., Desai Nihar R., Mosenzon Ofri, McGuire Darren K., Ray Kausik K., Leiter Lawrence A., Raz Itamar (2013). Saxagliptin and Cardiovascular Outcomes in Patients with Type 2 Diabetes Mellitus. New England Journal of Medicine.

[cit18] Go Alan S., Chertow Glenn M., Fan Dongjie, McCulloch Charles E., Hsu Chi-yuan (2004). Chronic Kidney Disease and the Risks of Death, Cardiovascular Events, and Hospitalization. New England Journal of Medicine.

[cit19] Tonelli Marcello, Muntner Paul, Lloyd Anita, Manns Braden J, Klarenbach Scott, Pannu Neesh, James Matthew T, Hemmelgarn Brenda R (2012). Risk of coronary events in people with chronic kidney disease compared with those with diabetes: a population-level cohort study. The Lancet.

[cit20] Bakris George L., Agarwal Rajiv, Anker Stefan D., Pitt Bertram, Ruilope Luis M., Rossing Peter, Kolkhof Peter, Nowack Christina, Schloemer Patrick, Joseph Amer, Filippatos Gerasimos (2020). Effect of Finerenone on Chronic Kidney Disease Outcomes in Type 2 Diabetes. New England Journal of Medicine.

